# Lung transplantation from donation after circulatory death donors over 55 years old: A national analysis of outcomes and utilization

**DOI:** 10.1016/j.jhlto.2025.100423

**Published:** 2025-11-01

**Authors:** Isaac S. Alderete, Samantha E. Halpern, Oliver K. Jawitz, Ahmed Gurses, Haoran Jiang, Hiroshi Date, Jacob Klapper, Matthew G. Hartwig, Kunal J. Patel

**Affiliations:** a Department of Surgery, Hospital of the University of Pennsylvania, Philadelphia, PA; b Department of Surgery, Massachusetts General Hospital, Boston, MA; c Department of Surgery, Duke University Medical Center, Durham, NC

**Keywords:** Donation after circulatory death, Lung transplantation, Older donors, Organ utilization, Organ Procurement Organization

## Abstract

**Background:**

Lungs from older donation after circulatory death (DCD) donors are underutilized; however, these organs may represent an opportunity to expand the donor pool. Herein, we evaluated utilization and outcomes of lung transplants using lungs from older DCD donors.

**Methods:**

Using national data from 2016 to 2024, we identified all adult DCD donors and corresponding isolated lung transplant recipients. Multivariable logistic regression was used to identify predictors of lung utilization. Kaplan-Meier and Cox proportional hazards methods were used to compare graft survival between recipients of lungs from DCD donors <55 and ≥55 years.

**Results:**

Among 10,769 older (age ≥ 55) DCD donors identified, lungs were transplanted from only 302 (2.8%) with significant organ procurement organization- and center-level variation in use. Just one center exceeded 50 transplants using older DCD donors over the study period. Utilization increased over time (7 cases in 2016 vs 111 in 2024; *p* < 0.001). Ex-vivo lung perfusion (odds ratio 5.93) and higher PaO₂/FiO₂ ratio (odds ratio 1.33 per 50-point increase) were independently associated with transplantation of older DCD donor lungs. One- and three-year graft survival were similar between age groups; older donor age was not associated with increased risk of graft failure in adjusted models.

**Conclusions:**

Lungs from older DCD donors remain underutilized despite comparable outcomes. Their use is highly concentrated among a small number of centers and organ procurement organizations, suggesting that local behaviors and infrastructure strongly influence disposition. Broader adoption of ex-vivo lung perfusion and strategic recipient matching may support safe expansion of this untapped donor pool.

## Background

Lung transplantation (LTx) remains the definitive therapy for patients with end-stage lung disease, but its use is limited by a persistent shortage of suitable donor lungs.^1^ As demand continues to outpace supply, transplant programs have increasingly turned to donation after circulatory death donors (DCD) to expand the pool.^2^, ^3^, ^4^, ^5^ Despite early concerns about primary graft dysfunction and ischemic injury, DCD lung use has increased steadily over the past decade, driven by accumulating evidence of comparable outcomes to donation after brain death (DBD).^3^

Donor age has historically been viewed as a limiting factor in LTx due to concerns about graft quality, increased comorbidities, and higher susceptibility to ischemic injury. While several studies have demonstrated that lungs from older donors can be used safely under select conditions, much of this work has focused on DBDs, with limited data addressing the use of older lungs in the context of DCDs.^6^, ^7^, ^8^ DCD donors already carry additional logistical and physiological challenges, and when coupled with older age, they represent a subgroup often perceived as nonideal. As a result, older DCD donors remain underutilized, despite advances in preservation and recipient management.^4^, ^9^ Although prior studies have explored utilization rates and selection patterns for DCD donors broadly, little is known about the current landscape of DCD lungs from older donors. It is unclear which centers or organ procurement organizations (OPOs) use these lungs, how utilization has changed over time, or whether outcomes differ compared to younger DCD donors.

Accordingly, we leveraged national transplant registry data to describe trends in the use of older DCD lungs, identify variation across OPOs and transplant centers, and compare short-term outcomes following transplantation. We hypothesized that lungs from older DCD donors result in comparable post transplant outcomes to those from younger donors, but that utilization is limited to a small number of OPOs and transplant centers.

## Methods

### Data source

We conducted a retrospective cohort study using data from the Scientific Registry of Transplant Recipients. The analysis period spanned January 1, 2016, through December 31, 2024, which was selected to capture the modern era of DCD LTx, including the introduction and uptake of ex-vivo lung perfusion and contemporary donor management practices. This study was approved by the Duke University Institutional Review Board (Pro00073879) and conducted in compliance with the ISHLT Ethics Statement.

### Study population

We included all adult-controlled DCD donors and excluded those with indeterminate lung disposition. Recipients were excluded if they underwent multiorgan transplantation, were pediatric patients, or had missing post transplant survival or follow-up data.

### Outcomes

The primary donor-level outcome was lung utilization, defined as transplantation of at least one lung. Additional donor metrics included rates of lung recovery and discard. The primary recipient-level outcome was 1-year graft survival, defined as a composite of death or re-transplantation within 1 year post transplant. Secondary outcomes included 30-day composite survival, 3-year composite survival, early post transplant extracorporeal membrane oxygenation (ECMO) use, prolonged mechanical ventilation, dialysis during the index hospitalization, intensive care unit readmission, and documented rejection episodes.

### Statistical analysis

Donor age was evaluated both as a continuous and categorical variable. The categorical threshold (≥55 years) was derived from an inflection point identified through restricted cubic spline modeling of donor age in relation to graft survival (Supplementary Figure S1). Donor and recipient characteristics were summarized descriptively and compared across age groups using chi-squared tests for categorical variables and Wilcoxon rank-sum tests for continuous variables. For donor-level analyses, we used multivariable logistic regression to identify predictors of lung utilization among DCD donors aged ≥55. To account for clustering and unmeasured center-level variation in procurement practices, a random intercept for OPO was included. Trends in the use of older DCD lungs over time were assessed using the Cochran-Armitage test for trend.

Recipient survival was estimated using Kaplan-Meier methods and log-rank testing. Cox proportional hazards models were constructed to assess the association between donor age group and one-year graft survival, adjusting for prespecified donor, recipient, and transplant characteristics based on prior literature and clinical relevance. To provide a more granular assessment, donor age was additionally modeled as a continuous variable in sensitivity analyses. To evaluate mid-term graft survival, we performed a subanalysis restricted to recipients transplanted between January 1, 2016, and December 31, 2021, to ensure complete three-year follow-up. Missingness among covariates was minimal and was addressed using median or mode imputation. Proportional hazards assumptions were evaluated using Schoenfeld residuals. All analyses were conducted in R version 4.3.0, with 2-sided *p*-value < 0.05 considered statistically significant.

## Results

### Donor and recipient baseline characteristics

**Figure 1:**
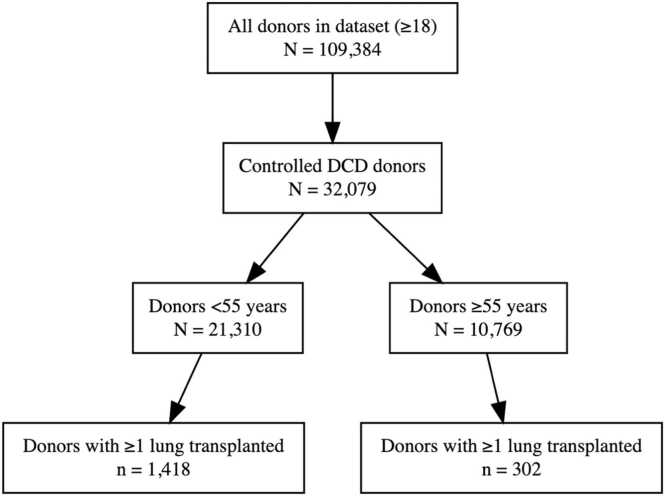
*Flow diagram of donor inclusion and lung transplantation (LTx) by donor age, 2016-2024.* Flowchart depicting derivation of the study cohort from all adult donors (≥18 years) in the national database between 2016 and 2024. Among 109,384 total donors during this time period, 32,079 were controlled donation-after-circulatory-death (DCD) donors and constituted the analytic cohort. Of these, 21,310 (66.4%) were aged <55 years and 10,769 (33.6%) were aged ≥55 years. Lungs from 1,418 younger donors (6.7%) and 302 older donors (2.8%) were transplanted.

Between 2016 and 2024, a total of 32,079 controlled DCD donors aged ≥18 years met inclusion criteria. Of these, 10,769 (33.6%) were aged ≥55 years and 21,310 (66.4%) were aged <55. Among donors aged ≥55 years, most were between 55 and 59 years (64%), with progressively fewer donors in each older age bracket (Supplementary Table S1). At the donor level, lungs were transplanted from 302 older donors (2.8%) and 1,418 younger donors (6.7%), yielding a total of 1,720 donors who donated at least one lung for transplant. Ex-vivo lung perfusion (EVLP) was used in 2.3% (*n* = 480) of eligible DCD donors <55 years and 0.9% (*n* = 93) of donors ≥55 years, with comparable EVLP-to-transplant conversion rates (59.8% vs 60.2%) (Figure 1).

One thousand six hundred six adult isolated lung transplant recipients were included in the post transplant outcomes analysis (*n* = 1,345 received lungs from donors <55; *n* = 261 from donors ≥55). Recipients of lungs from older donors were more likely to have blood type B (14.6% vs 9.1%, *p* = 0.045). Transplant year also differed significantly, with a greater proportion of older donor transplants occurring in more recent years (e.g., 29.1% in 2024 vs 18.6%; *p* = 0.005). Redo lung transplants were included and accounted for 2.2% (*n* = 36) of the total cohort, with similar proportions among recipients of donors <55 years (2.3%) and ≥55 years (1.9%). Donor cause of death differed significantly by age, with stroke more common among older donors and anoxia among younger donors (*p* < 0.001). No significant differences were observed in recipient age, sex, race/ethnicity, BMI, diagnosis group, hospitalization status, ECMO or ventilator use at transplant, ischemic time, or donor-recipient distance (Table 1).

**Table 1:** Baseline Characteristics of Transplant Recipients Stratified by Donor Age Group

Variable	<55 years	≥55 years	*p*-value
*n*	1,345	261	
Recipient age (years)	59.60 (11.18)	60.73 (11.31)	0.136
Recipient sex-female	538 (40.0)	119 (45.6)	0.107
Recipient ethnicity			0.641
White	1,063 (79.0)	197 (75.5)	
Black	127 (9.4)	28 (10.7)	
Hispanic	115 (8.6)	27 (10.3)	
Other	40 (3.0)	9 (3.4)	
Recipient BMI (kg/m²)	26.00 (4.38)	26.44 (4.53)	0.147
Recipient ABO type			**0.045**
A	516 (38.4)	101 (38.7)	
AB	35 (2.6)	6 (2.3)	
B	122 (9.1)	38 (14.6)	
O	672 (50.0)	116 (44.4)	
Recipient diagnosis group			
Group A	390 (29.0)	68 (26.1)	0.584
Group B	45 (3.3)	9 (3.4)	
Group C	51 (3.8)	7 (2.7)	
Group D	859 (63.9)	177 (67.8)	
Medical condition at listing			
ICU	169 (12.6)	29 (11.2)	0.585
Hospitalized, nonICU	126 (9.4)	29 (11.2)	
Not hospitalized	1,049 (78.1)	202 (77.7)	
ECMO at transplant	64 (4.8)	12 (4.6)	1.000
Ventilated at transplant	53 (3.9)	13 (5.0)	0.546
Donor age (years)	37.11 (10.21)	59.03 (3.48)	**<0.001**
Donor sex-female	543 (40.4)	122 (46.7)	0.065
Donor BMI (kg/m²)	27.89 (6.55)	28.51 (6.15)	0.159
Donor cause of death			**<0.001**
Anoxia	594 (44.2)	73 (28.0)	
Stroke	333 (24.8)	128 (49.0)	
Head trauma	381 (28.3)	56 (21.5)	
CNS tumor	1 (0.1)	0 (0.0)	
Other	36 (2.7)	4 (1.5)	
Ischemic time (hours)	8.85 (4.42)	8.81 (4.10)	0.891
Donor-recipient distance (nm)	317.28 (334.01)	334.19 (358.93)	0.460
Transplant type			0.180
Double	1,128 (83.9)	208 (79.7)	
Left	103 (7.7)	22 (8.4)	
Right	114 (8.5)	31 (11.9)	
Transplant year			**0.005**
2016	79 (5.9)	8 (3.1)	
2017	73 (5.4)	11 (4.2)	
2018	90 (6.7)	17 (6.5)	
2019	128 (9.5)	20 (7.7)	
2020	164 (12.2)	19 (7.3)	
2021	158 (11.7)	27 (10.3)	
2022	161 (12.0)	32 (12.3)	
2023	242 (18.0)	51 (19.5)	
2024	250 (18.6)	76 (29.1)	
UNOS region			0.110
1	86 (6.4)	14 (5.4)	
2	104 (7.7)	17 (6.5)	
3	105 (7.8)	21 (8.0)	
4	116 (8.6)	24 (9.2)	
5	210 (15.6)	46 (17.6)	
6	15 (1.1)	2 (0.8)	
7	89 (6.6)	18 (6.9)	
8	43 (3.2)	4 (1.5)	
9	87 (6.5)	24 (9.2)	
10	317 (23.6)	74 (28.4)	
11	173 (12.9)	17 (6.5)	

ABO, blood group classification; BMI, body mass index; ECMO, extracorporeal membrane oxygenation; ICU, Intensive care unit; UNOS, United Network for organ sharing.

Bold values indicates statistically significant value for *P*<0.05.

### Donor lung disposition and utilization by age

Disposition of each lung was tracked, with donors potentially contributing up to 2 lungs each (Table 2). From younger donors, 2,687 lungs (15.9%) were transplanted, compared to just 558 lungs (6.5%) from older donors. Compared with younger donors, older donors had a higher rate of lungs not recovered (83.2% vs 75.8%), but comparable rates of consent attainment, not requested, and discard. Among reasons for lack of donor allograft recovery, poor organ function was the most cited reason in both younger and older donors (26.7% vs 26.4%). Diseased organs were cited more frequently in older donors compared to younger donors (18.4% vs 10.1%). All other reasons for non-recovery are summarized in Table 3.

**Table 2:** Lung Disposition by Donor Age Group

Reason for lung disposition	<55	≥55
Authorization not requested	62 (0.4%)	75 (0.9%)
Authorization not obtained	328 (1.9%)	206 (2.4%)
Organ not recovered	12,791 (75.8%)	7,169 (83.2%)
Recovered not for Tx	744 (4.4%)	511 (5.9%)
Recovered for Tx but not Tx	268 (1.6%)	93 (1.1%)
Transplanted	2,687 (15.9%)	558 (6.5%)

This data is tabulated as per lung, meaning each donor can donate up to 2 lungs.

**Table 3:** Reasons for Lung Non-Recovery in Older vs Younger DCD Donors

Reason for organ non-recovery	<55	≥55
Poor organ function	3,407 (26.7%)	1,890 (26.4%)
Diseased organ	1,288 (10.1%)	1,318 (18.4%)
Other specify	1,463 (11.5%)	1,006 (14.1%)
Time constraints	1,348 (10.6%)	538 (7.5%)
Organ refused by all national programs	1,155 (9%)	527 (7.4%)
Donor medical history	821 (6.4%)	702 (9.8%)
PO2 < 200 on O2 challenge	879 (6.9%)	386 (5.4%)
Infection	426 (3.3%)	309 (4.3%)
No recipient located	507 (4%)	134 (1.9%)
Hemodynamically unstable donor	528 (4.1%)	112 (1.6%)
Trauma to organ	238 (1.9%)	48 (0.7%)
Organ refused by all regional programs	175 (1.4%)	55 (0.8%)
Ruled out after evaluation in OR	128 (1%)	24 (0.3%)
Medical examiner restricted	122 (1%)	12 (0.2%)
Cardiac arrest	59 (0.5%)	23 (0.3%)
Refused by all programs with urgent need	67 (0.5%)	6 (0.1%)
Donor social history	39 (0.3%)	31 (0.4%)
No potential recipients on match run	40 (0.3%)	14 (0.2%)
Positive hepatitis	32 (0.3%)	10 (0.1%)
Positive HIV	28 (0.2%)	2 (0%)
Anatomical abnormalities	7 (0.1%)	3 (0%)
Positive gram stain	6 (0%)	2 (0%)
Vascular damage	2 (0%)	2 (0%)
Biopsy findings	2 (0%)	0 (0%)
No local recovery team	6 (0%)	0 (0%)
Surgical damage in OR	1 (0%)	0 (0%)

Among 19,960 lungs labeled as “Organ Not Recovered,” 32 lungs (17 from donors <55, 15 from ≥55) lacked reason codes and were excluded.

HIV, human immunodeficiency virus.

### OPO- and center-level variation in older DCD lung use

**Figure 2:**
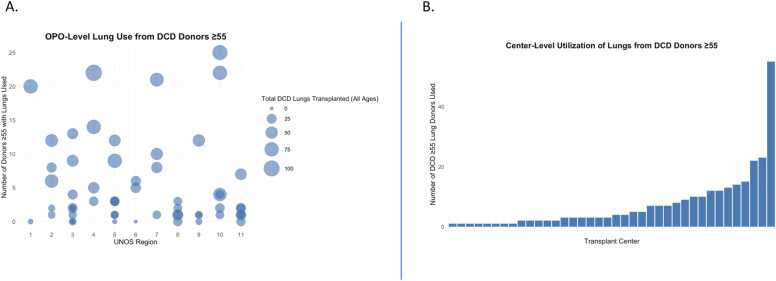
*OPO- and center-level variation in the use of lungs from older DCD donors, 2016-2024*. (A) OPO-level bubble plot displaying the number of DCD donors aged ≥55 with at least one lung used for transplant by UNOS region. Bubble size represents the total number of DCD lung transplants performed (all donor ages) by each OPO during the study period. (B) Histogram showing the number of DCD donors aged ≥55 with lungs used by each transplant center. Each bar represents one center, ranked by volume.

Among 57 OPOs included in the study, 49 recovered older DCD donor lungs that were ultimately transplanted. The median number of transplants per OPO was 3 (IQR: 1-9), with a maximum of 25 transplants. Notably, only 5 OPOs facilitated the transplant of ≥20 lungs from older donors during the study period (Figure 2A).

**Figure 3:**
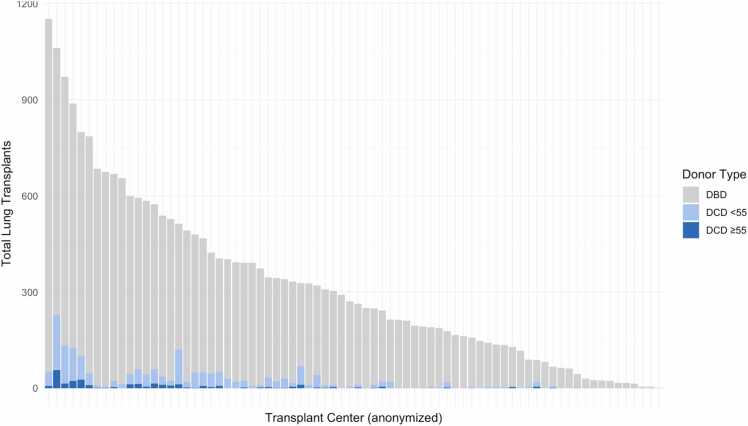
*Center-level total lung transplant volume and DCD donor composition, 2016-2024.* Bars represent total lung transplants performed by each U.S. transplant center during the study period, stratified by donor type: donation-after-brain-death (DBD, gray), DCD donors <55 years (light blue), and DCD donors ≥55 years (dark blue).

Across 38 transplant centers that utilized lungs from older DCD donors, the median number of transplants per center was 3.5 (IQR: 1-9). The highest center volume was 55 transplants, with only one center performing more than 50 older DCD lung transplants over the study period (Figure 2B). To further contextualize these findings, center-level total lung-transplant volumes during the study period are shown in Figure 3, stratified by donor type. Only a small fraction of overall activity involved DCD donors, and use of older (≥55 years) DCD lungs was almost entirely limited to high-volume programs.

### Temporal trends in utilization of older DCD lungs

**Figure 4:**
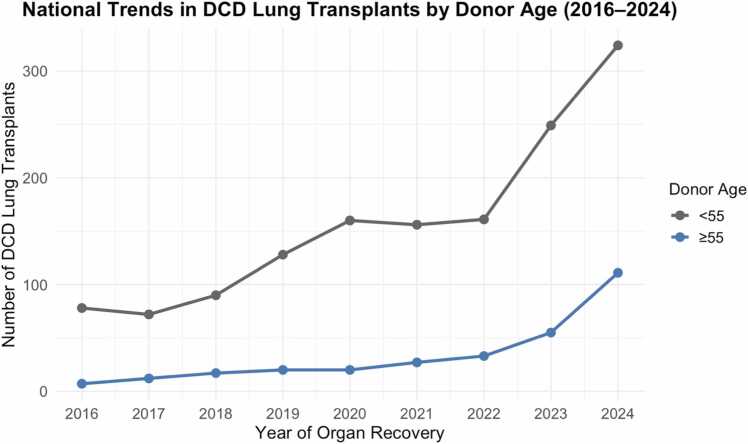
*National trends in DCD LTx by donor age, 2016-2024.* Annual number of DCD lung transplants performed using donors aged <55 (gray) versus ≥55 years (blue). While total DCD lung transplant volume has increased steadily over the study period, use of older DCD donors remains disproportionately low.

Between 2016 and 2024, the number of DCD lung transplants from donors aged ≥55 increased from 7 to 111, while transplants from donors <55 increased from 78 to 324 (Figure 4). The proportion of annual DCD lung transplants derived from donors ≥55 rose from 8.2% in 2016 to 25.5% in 2024. A Cochran-Armitage test for trend was performed to assess for a statistically significant change in the proportion of older donor use over time, demonstrating a significant upward trend in the proportion of transplanted DCD lungs from donors aged ≥55 compared to those <55 across the study period (*p* < 0.001).

### Predictors of older DCD lung utilization

In a mixed-effects logistic regression model adjusting for donor and transplant characteristics and including a random intercept for OPOs, several factors were independently associated with the likelihood of lung utilization among older DCD donors (Table 4). Increasing donor age was associated with lower odds of lung use (adjusted OR per 5-year increase: 0.60, 95% CI: 0.49-0.74, *p* < 0.001), as was a donor history of cigarette use (OR: 0.22, 95% CI: 0.14-0.36, *p* < 0.001). Male donor sex was also associated with reduced utilization compared to females (OR: 0.72, 95% CI: 0.53-0.98, *p* = 0.034). In contrast, a higher donor PaO₂/FiO₂ ratio significantly increased the odds of utilization (OR per 50-point increase: 1.33, 95% CI: 1.26-1.41, *p* < 0.001), as did the use of EVLP prior to transplant (OR: 5.93, 95% CI: 3.44-10.2, *p* < 0.001).

**Table 4:** Predictors of Lung Utilization From DCD Donors Aged ≥55 Years

Variable	Adjusted OR [95% CI]	*p*-value
Donor age (per 5 years)	0.60 [0.49-0.74]	**<0.001**
B blood type (A as reference)	0.82 [0.50-1.35]	0.440
O blood type (A as reference)	0.89 [0.64-1.23]	0.476
Donor smoking history	0.22 [0.13-0.36]	**<0.001**
Male sex	0.72 [0.53-0.98]	0.034
BMI (per unit)	0.98 [0.95-1.00]	0.059
P/F ratio (per 50 units)	1.33 [1.26-1.41]	**<0.001**
Ex-vivo lung perfusion	5.93 [3.44-10.22]	**<0.001**

BMI, body mass index; DCD, donation after circulatory death.

Bold values indicates statistically significant value for *P*<0.05.

### Post transplant outcomes

Recipients of lungs from donors aged ≥55 years experienced slightly higher rates of early post transplant complications compared to those receiving lungs from younger donors. ECMO use at 72 hours was more common in the ≥55 group (20% vs 15%; *P* = 0.025), and median hospital length of stay was longer (29.0 days [IQR: 18.0-55.0] vs 23.0 days [15.0-40.0]; *p* < 0.001). Prolonged intubation at 72 hours was also more frequent among recipients of older donor lungs (48% vs 40%; *p* = 0.028). Rates of dialysis, acute rejection prior to discharge, and treatment for rejection within 1 year were similar between groups.

**Figure 5:**
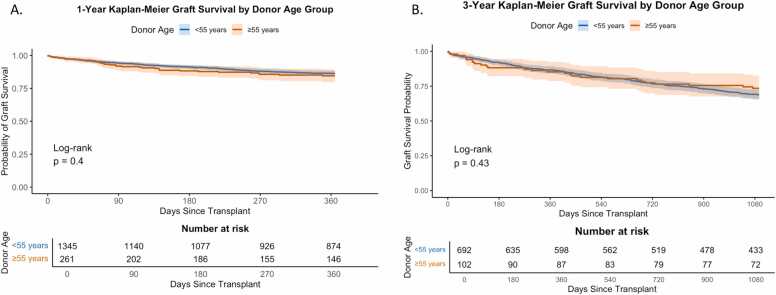
Kaplan-Meier graft survival following LTx from DCD donors <55 vs ≥55 Years of Age. (A) One-year graft survival was similar between recipients of lungs from donors <55 years and those ≥55 years (log-rank *p* = 0.40). (B) Among recipients with complete follow-up to 3 years, graft survival remained comparable by donor age group (log-rank *p* = 0.43). Shaded bands represent 95% confidence intervals.

In unadjusted Kaplan-Meier analysis, 30-day composite graft survival was nearly identical: 97.2% (95% CI: 96.3-98.1) for donors <55% and 97.2% (95% CI: 95.2-99.3) for donors ≥55 (*p* = 0.983). One-year survival was also comparable: 86.4% (95% CI: 84.4-88.3) for <55% and 84.5% (95% CI: 79.7-89.5) for ≥55 (*p* = 0.403) (Figure 5A). In an adjusted model, increasing recipient age was associated with higher 1-year mortality (HR 1.15, 95% CI: 1.06-1.25; *p* = 0.001), as was longer ischemic time (HR 1.04, 95% CI: 1.01-1.08; *p* = 0.015). Recipients with blood type B had higher risk compared to those with blood type A (HR 1.76, 95% CI: 1.12-2.78; *p* = 0.015). Importantly, donor age ≥55 years was not significantly associated with 1-year mortality. Full results are shown in Table 5. To evaluate whether dichotomizing donor age masked potential nonlinear effects, a sensitivity analysis was performed modeling donor age as a continuous variable. Donor age remained as a nonsignificant predictor of 1-year mortality (HR 1.04 per 5-year increase, 95% CI: 0.98-1.10; *p* = 0.21), consistent with the primary model.

**Table 5:** Cox Proportional Hazards Model for Predictors of 1-Year Post Transplant Mortality

Variable	Hazard ratio (95% CI)	*p*-value
Donor age ≥ 55	1.11 [0.77-1.62]	0.570
Recipient age (per 5 years)	1.15 [1.06-1.25]	**0.001**
Male recipient	0.73 [0.53-1.01]	0.057
Black race/ethnicity (vs white)	1.49 [0.96-2.33]	0.077
Hispanic race/ethnicity (vs white)	1.12 [0.67-1.86]	0.668
Other race/ethnicity (vs white)	1.55 [0.74-3.23]	0.242
Recipient BMI	1.01 [0.97-1.04]	0.743
AB blood type (A as reference)	1.75 [0.79-3.93]	0.170
B blood type (A as reference)	1.76 [1.12-2.78]	**0.015**
O blood type (A as reference)	1.23 [0.89-1.70]	0.200
Disease Group B (Group A as reference)	1.20 [0.51-2.84]	0.675
Disease Group C (Group A as reference)	1.64 [0.62-4.34]	0.317
Disease Group D (Group A as reference)	1.28 [0.90-1.84]	0.174
Hospitalized, nonICU (vs ICU)	0.95 [0.50-1.80]	0.874
Not hospitalized (vs ICU)	0.72 [0.43-1.22]	0.222
Recipient on ECMO	1.41 [0.65-3.03]	0.384
Recipient on ventilator	1.17 [0.54-2.53]	0.686
Male donor	1.22 [0.88-1.69]	0.224
Donor BMI	1.00 [0.97-1.02]	0.711
Ischemic time (hours)	1.04 [1.01-1.08]	**0.015**
EVLP use	1.11 [0.79-1.56]	0.562

Bold values indicates statistically significant value for *P*<0.05.

To evaluate longer-term outcomes, we conducted a subanalysis restricted to recipients transplanted between 2016 and 2021 to ensure complete 3-year follow-up (*n* = 794). In unadjusted Kaplan-Meier analysis, three-year graft survival was comparable between age groups: 76.4% (95% CI: 73.1-79.8) for donors <55% and 78.2% (95% CI: 69.7-87.7) for donors ≥55 (*p* = 0.43; Figure 5B). In the corresponding multivariable Cox proportional hazards model, donor age ≥55 was not significantly associated with increased risk of graft failure at 3 years (adjusted HR 0.83, 95% CI: 0.55-1.25; *p* = 0.38). Full model results are presented in Supplementary Table S2.

## Discussion

In this national study of DCD LTx, we found that lungs from only 2.8% of older DCD donors were used for transplantation. Despite this low utilization rate, 1-year and 3-year graft survival among recipients of lungs from older donors was comparable to those from younger donors. Older DCD lung use was strongly associated with use of EVLP, suggesting that EVLP may be a key enabler of safe expansion into this donor population. Use of older DCD lungs remained highly concentrated within a limited number of OPOs and transplant centers, underscoring substantial geographic and institutional variability in practice. These findings highlight both the safety and untapped potential of older DCD lungs and the need for broader utilization nationwide.

Historically, donor age has been a key exclusion criterion in LTx, particularly for DCD donors, where warm ischemia was presumed to compound age-related pulmonary frailty. However, our findings challenge this assumption, demonstrating that DCD donors aged ≥55 years can yield comparable post transplant outcomes to younger DCD donors when appropriately selected. These results mirror and extend findings from the DBD literature, including prior studies reporting noninferior mid- and long-term outcomes in recipients of lungs from donors ≥65 and ≥70 years.^6^, ^7^ While the latter study included only 69 recipients, our national DCD cohort demonstrated similar survival at both 1 and 3 years, reinforcing the generalizability of these findings. To our knowledge, this is the first national study to evaluate the impact of older donor age on outcomes in the context of DCD LTx. More recently, it has also been shown—using a combined cohort of DBD and DCD donors—that accepting lungs from older donors may offer survival benefit in select high-acuity recipients.^10^ Collectively, these findings support the broader use of older DCD lungs and suggest that donor age, when considered in isolation, may be an overly conservative barrier to utilization—particularly as tools like EVLP and improvements in donor management continue to evolve.

Despite comparable outcomes, we observed variation in the use of older DCD lungs across transplant centers and OPOs. Only a minority of centers performed more than a handful of these transplants, and just one exceeded 50 over the entire study period. Similarly, while most OPOs facilitated at least 1 transplant using an older DCD donor, the vast majority contributed very few. This degree of variability suggests that decisions around recovery and implantation are often driven more by local culture, perceived risk, and logistical factors than by standardized clinical criteria. Notably, in our study, the majority of older DCD lung transplants occurred at high-volume centers, which likely contributes to the comparable outcomes observed in this cohort. These programs likely possess greater institutional experience, multidisciplinary coordination, and familiarity with extended-criteria donor management, all of which can mitigate risks associated with older donors. Further, previous studies have demonstrated that OPO-level behaviors—including consent acquisition and willingness to pursue nonideal donors—substantially shape the donor pool and influence transplant potential.^6^, ^11^, ^12^ In this context, older DCD lungs may represent an underutilized resource whose disposition is governed less by donor quality than by the environment in which the offer is made. Our finding that EVLP was independently associated with nearly a six-fold increase in utilization underscores its value in expanding the effective donor pool. In the context of this study, it is unclear whether EVLP rehabilitated physiologically marginal lungs or simply enabled transplant teams to evaluate and commit to organs that might otherwise have been declined due to donor age alone.^13^, ^14^, ^15^ Either mechanism supports its use as a practical tool for increasing utilization. Given the resource intensity of EVLP, its impact is likely maximized at high-volume, well-resourced centers with established workflows and clinical judgment to interpret perfusion data in real-time.^16^ Rather than serving as a universal solution, EVLP may be best viewed as a strategic asset for placing extended-criteria lungs—including those from older DCD donors—when paired with institutional capacity and experience.

In select cases, allocation out of sequence (AOOS) may serve as a mechanism to facilitate the use of older DCD lungs that are otherwise unlikely to be recovered. AOOS allows an OPO to offer an organ directly to a transplant center outside of the standard match run when it is unlikely to be placed through traditional allocation pathways. This process is typically used when all centers on the match run have declined the organ, or when time-sensitive factors—such as donor instability, prolonged ischemic time, or logistical barriers—make sequential offers impractical. In our study, most of these organs were never procured, despite our finding of comparable outcomes to younger donor lungs at both 1 and 3 years. This pattern suggests that many viable grafts are bypassed upstream—not due to recipient ineligibility or graft failure, but rather due to institutional hesitancy or logistical barriers. AOOS offers a pragmatic tool in this context, allowing expedited allocation to centers with demonstrated interest and capacity to evaluate and accept older DCD lungs.^17^, ^18^ While AOOS has raised concerns around equity and transparency,^19^, ^20^ its selective use—guided by objective criteria such as center experience or historical acceptance—could help ensure that transplantable organs are not lost to system inertia. As with EVLP, AOOS is not a universal fix but may be a practical adjunct to expand access and reduce missed opportunities within an already limited donor pool.

Recipient selection likely plays a meaningful role in the observed outcomes when using older DCD donors. While our findings show comparable outcomes, they likely reflect selective matching—where centers willing to use these grafts choose recipients perceived to be lower risk. Importantly, our analysis suggests that this caution may be justified: older recipient age was independently associated with increased mortality at both 1 and 3 years, and recipients who were not hospitalized at the time of transplant had significantly better 3-year survival than those listed in the intensive care unit. These data support the use of older DCD lungs in younger, clinically stable candidates, particularly during early adoption. That said, this should not be interpreted as a contraindication for use in older recipients; prior studies in DBD transplantation have shown that age-matching in DBD donors—older donors to older recipients—does not necessarily compromise outcomes,^21^ and in some cases matching older lungs to younger recipients is associated with worse outcomes.^22^, ^23^ Moreover, in our dataset, non-recovery of older DCD lungs was rarely due to an inability to place the organ, suggesting that low utilization may be more a reflection of procurement practices than a true lack of suitable recipients.

This study has several limitations. As with all registry analyses, our findings are subject to limitations in data granularity. Important donor- and recipient-level variables—such as agonal time, extubation-to-arrest interval, or intraoperative ventilatory strategies—are not well captured in the Scientific Registry of Transplant Recipient dataset but likely influence outcomes in DCD LTx. Second, although we adjusted for key clinical covariates, unmeasured confounding may persist in our survival models. Additionally, the generalizability of our findings to international settings is uncertain, as countries with established DCD programs have not specifically reported outcomes among older DCD donors, though similar patterns would likely be expected in high-volume, experienced centers. Furthermore, uncontrolled DCD donors were excluded from this analysis, which may limit the generalizability of our findings to all forms of DCD practice. Finally, data on EVLP were limited to binary indicators of use; specific perfusion parameters, protocols, and decision-making processes were unavailable, limiting our ability to disentangle the mechanism by which EVLP was associated with increased utilization. Nonetheless, this study represents the most comprehensive national analysis to date of older DCD lung utilization and outcomes in the United States.

## Conclusion

In this national analysis of DCD LTx, we found that lungs from donors aged ≥55 years remain underutilized despite demonstrating comparable early and mid-term outcomes to those from younger donors. While most centers and OPOs have engaged in the use of older DCD lungs, clinical activity remains concentrated in a small subset of programs. Utilization was strongly associated with the use of ex vivo lung perfusion, highlighting its role as a practical tool in extended-criteria donation. Our data also suggest that careful recipient selection—favoring younger, clinically stable candidates—may support safe adoption of older DCD lungs. Together, these findings support broader consideration of older DCD donors as a viable source of transplantable lungs and underscore the importance of institutional practices and clinical infrastructure in shaping donor utilization.

## Conflicts of Interest statement

The authors declare the following financial interests/personal relationships, which may be considered as potential competing interests: Matthew Hartwig reports a relationship with Paragonix Technologies, Inc. that includes consulting or advisory. Matthew Hartwig reports a relationship with TransMedics Inc. that includes consulting or advisory. If there are other authors, they declare that they have no known competing financial interests or personal relationships that could have appeared to influence the work reported in this paper.

## Financial support

The authors received no funding for this study.

## Footnotes

Appendix A Supplementary data associated with this article can be found in the online version at doi:10.1016/j.jhlto.2025.100423.
