# Supplementary material for: Lung transplantation from donation after circulatory death donors over 55 years old: A national analysis of outcomes and utilization
Source: JHLT Open. 2025 Nov 1;11:100423. doi: 10.1016/j.jhlto.2025.100423 (PMC12677173; doi:10.1016/j.jhlto.2025.100423)
Supplement: Supplementary file 1 — Supplementary material [file mmc1.docx]

**Table of Contents**

| **Section** | **Page** |
| --- | --- |
| Supplemental Figure S1. Association Between Donor Age and Risk of 1-Year Graft Failure | 2 |
| Supplemental Table S1. Donor Demographics by 5-Year Age Group (≥55 Years) | 3 |
| Supplemental Table S2. Cox Proportional Hazards Model for 3-Year Mortality | 5 |

##
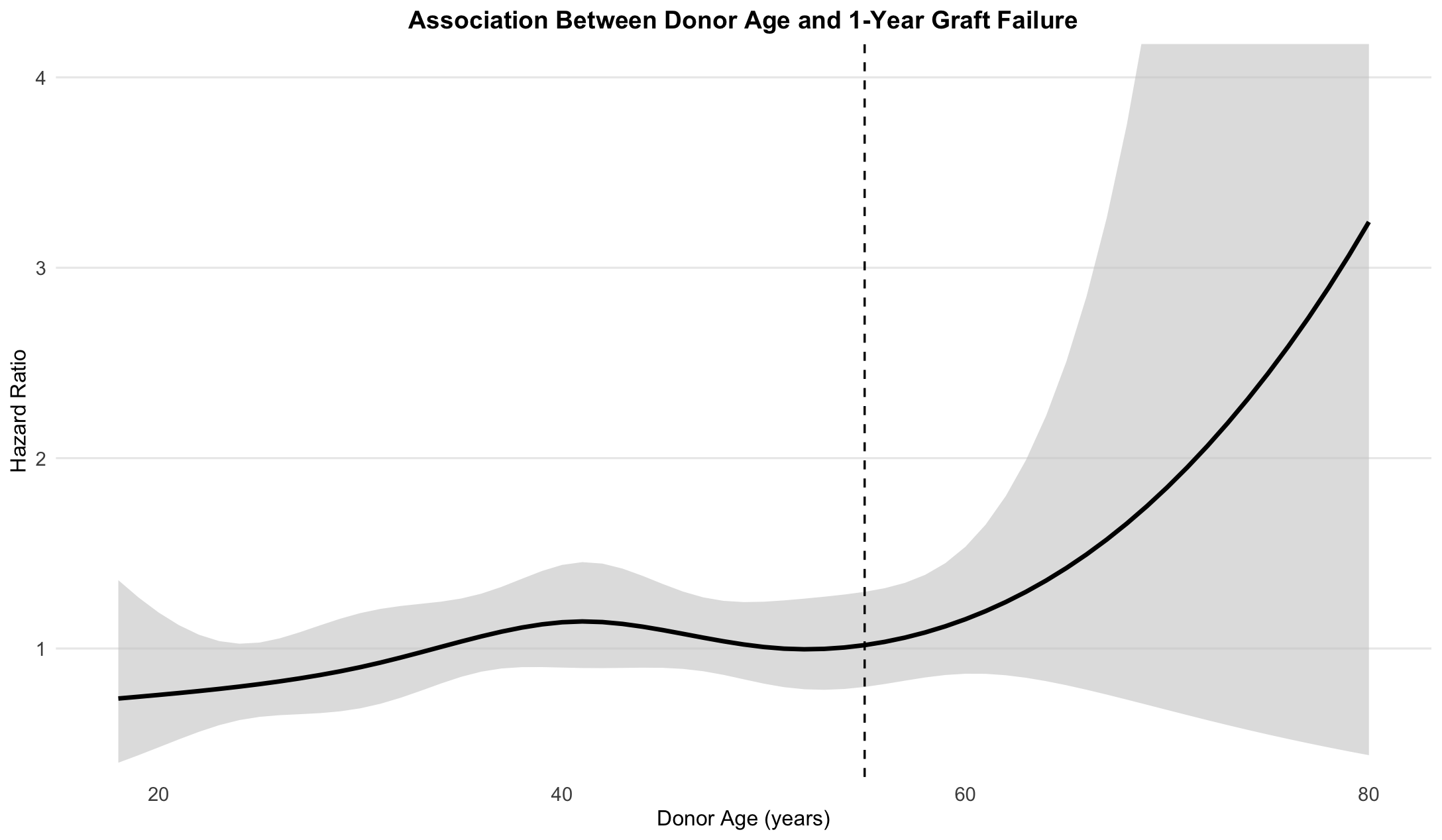


## *Supplemental Figure 1. Association Between Donor Age and Risk of 1-Year Graft Failure Among Adult Lung Transplant Recipients, 2016–2024.* A Cox proportional hazards model with a restricted cubic spline function (4 degrees of freedom) was used to evaluate the association between donor age and the hazard of 1-year graft failure among adult lung transplant recipients between 2016 and 2024. The solid line represents the estimated hazard ratio across the continuous range of donor age. A vertical dashed line is shown at age 55 to highlight a visual inflection point in risk and the cutoff used in subsequent stratified analyses.

## Supplemental Table 1. Donor Demographics by 5-Year Age Group (≥55 Years)

| Variable | 55–59 | 60–64 | 65–69 | 70+ | p-value |
| --- | --- | --- | --- | --- | --- |
| n | 167 | 74 | 18 | 2 |  |
| Donor age (years) | 56.93 (1.51) | 61.54 (1.32) | 66.78 (1.48) | 71.50 (2.12) | <0.001 |
| Male Donor | 95 (56.9) | 37 (50.0) | 6 (33.3) | 1 (50.0) | 0.253 |
| Donor BMI | 28.67 (5.71) | 29.02 (7.10) | 24.38 (4.47) | 33.37 (0.28) | 0.018 |
| Donor Cause of Death |  |  |  |  | 0.540 |
| Anoxia | 49 (29.3) | 21 (28.4) | 2 (11.1) | 1 (50.0) |  |
| Stroke | 79 (47.3) | 38 (51.4) | 11 (61.1) | 0 (0.0) |  |
| Head Trauma | 36 (21.6) | 15 (20.3) | 4 (22.2) | 1 (50.0) |  |
| Other | 3 (1.8) | 0 (0.0) | 1 (5.6) | 0 (0.0) |  |
| Donor History of Cigarette Use |  |  |  |  | 0.476 |
| No | 151 (90.4) | 67 (90.5) | 16 (88.9) | 2 (100.0) |  |
| Unknown | 1 (0.6) | 3 (4.1) | 0 (0.0) | 0 (0.0) |  |
| Yes | 15 (9.0) | 4 (5.4) | 2 (11.1) | 0 (0.0) |  |
| Donor History of Diabetes |  |  |  |  | 0.533 |
| No | 131 (78.4) | 60 (81.1) | 17 (94.4) | 1 (50.0) |  |
| Unknown | 2 (1.2) | 2 (2.7) | 0 (0.0) | 0 (0.0) |  |
| Yes | 34 (20.4) | 12 (16.2) | 1 (5.6) | 1 (50.0) |  |
| Ex-Vivo Lung Perfusion Used | 31 (19.5) | 17 (23.6) | 3 (16.7) | 0 (0.0) | 0.751 |

**Supplemental table 2. Cox Proportional Hazards Model for Predictors of 3-Year Post-Transplant Mortality. The model was restricted to patients transplanted between 2016 and 2021 to ensure complete 3-year follow-up.**

| Variable | Hazard Ratio (95% CI) | P-value |
| --- | --- | --- |
| Donor Age ≥ 55 | 0.83 [0.55 – 1.25] | 0.380 |
| Recipient Age (per 5 years) | 1.19 [1.10 – 1.29] | **<0.001** |
| Male Recipient | 0.94 [0.70 – 1.27] | 0.706 |
| Black race/ethnicity (vs White) | 1.54 [1.02 – 2.33] | **0.040** |
| Hispanic race/ethnicity (vs White) | 1.16 [0.67 – 2.02] | 0.588 |
| Other race/ethnicity (vs White) | 1.95 [0.97 – 3.92] | 0.061 |
| Recipient BMI | 1.00 [0.97 – 1.03] | 0.865 |
| AB blood type (A as reference) | 1.14 [0.56 – 2.31] | 0.721 |
| B blood type (A as reference) | 1.14 [0.71 – 1.84] | 0.582 |
| O blood type (A as reference) | 1.10 [0.83 – 1.46] | 0.502 |
| Disease Group B (Group A as reference) | 1.39 [0.68 – 2.87] | 0.370 |
| Disease Group C (Group A as reference) | 1.74 [0.81 – 3.76] | 0.157 |
| Disease Group D (Group A as reference) | 0.90 [0.65 – 1.25] | 0.532 |
| Hospitalized, non-ICU (vs ICU) | 0.81 [0.44 – 1.49] | 0.497 |
| Not Hospitalized (vs ICU) | 0.58 [0.36 – 0.92] | **0.022** |
| Recipient on ECMO | 1.28 [0.66 – 2.47] | 0.461 |
| Recipient on Ventilator | 0.96 [0.49 – 1.88] | 0.909 |
| Male Donor | 1.00 [0.74 – 1.34] | 0.983 |
| Donor BMI | 1.00 [0.98 – 1.02] | 0.847 |
| Ischemic Time (hours) | 1.05 [1.01 – 1.08] | **0.009** |
